# Genome Sequence Analysis of a G2P[4] Group A Rotavirus Strain with an Unusual Canine-Origin NSP1 A15 Genotype

**DOI:** 10.1128/genomeA.01315-17

**Published:** 2017-12-14

**Authors:** Chung-Chan Lee, Shih-Yen Chen, Chi-Neu Tsai, Cheng-Hsun Chiu

**Affiliations:** a Molecular Infectious Disease Research Center, Chang Gung Memorial Hospital, Taoyuan City, Taiwan; b Division of Pediatric Gastroenterology, Department of Pediatrics, Chang Gung Memorial Hospital, Chang Gung University College of Medicine, Taoyuan City, Taiwan; c Graduate Institute of Clinical Medical Sciences, Chang Gung University, Taoyuan City, Taiwan; d Department of Pediatrics, Chang Gung Memorial Hospital, Taoyuan City, Taiwan

## Abstract

Here, we disclose the complete genomic sequence of a rare rotavirus group A G2P[4]-I2-R2-C2-M2-A15-N2-T2-E2-H2 strain detected in a fecal specimen from a rotaviral acute gastroenteritis patient who had previously received the Rotarix vaccine.

## GENOME ANNOUNCEMENT

Rotaviruses are the major etiological agents of acute gastroenteritis (AGE) in young children and adults (1). The genome of rotavirus group A (RVA) consists of two outer capsid proteins, VP7 (glycoprotein; G protein) and VP4 (protease sensitive; P protein), which differentiate rotaviruses according to a binary classification system based on G and P genotypes and elicit neutralizing antibodies (2). Rotaviruses possess an RNA genome composed of 11 double-stranded segments encoding six structural proteins (VPs) and five nonstructural proteins (NSPs). The genome is assigned the complete descriptor of G*x*-P[*x*]-I*x*-R*x*-C*x*-M*x*-A*x*-N*x*-T*x*-E*x*-H*x* for VP7-VP4-VP6-VP1-VP2-VP3-NSP1-NSP2-NSP3-NSP4-NSP5, respectively, with *x* indicating the numbers of corresponding genotypes, which evolved from RNA fragments of rotaviruses that infect other species (3). Human RVA G2P[4] strains represent a genotype that has been one of major prominence in the past few years in Taiwan (4).

One child had been immunized with Rotarix (RVA/Vaccine/USA/Rotarix-A41CB052A/1988/G1P1A[8]) before the age of 1 year, and then got AGE caused by rotavirus at the age of 5 years. A fecal specimen from this patient (CCH764) was collected with the approval of the Institutional Review Board of the Chang Gung Memorial Hospital (CGMH) in Linkou, Taiwan (IRB-97-2465B). The RNA was extracted, treated with RNase-free DNase (Qiagen), and converted into a cDNA library following a reverse-transcription reaction performed according to the manufacturer recommendations (Illumina, San Diego, CA, USA). Further treatment with two rounds of duplex-specific nuclease (DSN) was performed on the cDNA libraries to reduce abundant rRNAs. The cDNA libraries were sequenced on an Illumina HiSeq2000 sequencing system to obtain a total of 68,495,460 reads, and after quality trimming with an average length 101 bp, a final 67,518,483 reads were obtained. Among the 67,518,483 reads, 66,580,152 reads were aligned to the human genome; the remaining 938,331 reads were further subjected to *de novo* assembly. Finally, 11 contigs were obtained and subjected to a BLAST search with RVA reference strains (5, 6). To confirm the sequencing results and fill the gaps, each RNA fragment was confirmed via PCR following direct sequencing via an ABI 3770 DNA sequencer. The complete genome sequence contained 11 full-length RNA fragments of RVAs. This RVA strain was designated RVA/Human-wt/TW/CCH764/2011/G2P[4] and closely resembles the DS-1-like strain, with 98% identity in both VP7 and VP4 sequences.

Among these 11 RNA fragments, the NSP1 gene of RVA/Human-wt/TW/CCH764/2011/G2P[4] is closely linked to an unusual genotype, A15 (RVA/Human-tc/ITA/PA260-97/1997/G3P[3]), which is a reassorting virus between feline, canine, and human rotaviruses from the Cat97-like and AU-1-like strains (7). The reassortment of NSP1 in this case indicates the tendency of RNA viruses to exchange genetic material frequently (8). Interestingly, the role of NSP1 in modulating host innate immunity or contributing to immune escape (9) and whether the A15 genotype NSP1 in RVA/Human-wt/TW/CCH764/2011/G2P[4] contributes to vaccine escape remain to be further elucidated.

### Accession number(s).

The nucleotide sequences of RVA/Human-wt/TW/CCH764/2011/G2P[4] determined in this study have been deposited in the GenBank database under accession numbers KP771726 and KX082721 for VP7 and VP4, respectively. The remaining GenBank accession numbers are KU925779 (NSP1), KU925780 (NSP2), KU925781 (NSP3), KU925782 (NSP4), KU925783 (NSP5), KU925784 (VP1), KU925785 (VP2), KU925786 (VP3), and KU925787 (VP6).
